# Diffusion tensor imaging and MR spectroscopy in the evaluation of neonatal encephalopathies

**DOI:** 10.1177/19345798251380181

**Published:** 2025-09-22

**Authors:** Adya Mehra, Priya Pattath Sankaran, Sheila S. Mathai, Rajagopal Kadavigere, Prakashini Koteshwara

**Affiliations:** 1 Department of Radiodiagnosis and Imaging, Kasturba Medical College Manipal, Manipal Academy of Higher Education, Manipal, India; 2 Department of Neonatology, Kasturba Medical College Manipal, Manipal Academy of Higher Education, Manipal, India

**Keywords:** diffusion tensor imaging, fractional anisotropy, hypoxic-ischemic encephalopathy, magnetic resonance spectroscopy, neonatal encephalopathy

## Abstract

**Background:**

Neonatal encephalopathy is a broad term encompassing many underlying pathologies, most commonly hypoxic-ischemic encephalopathy. Though this diagnosis has long been derived from relevant history, examination and lab parameters, this study aims to illustrate the diagnostic power of advanced radiological techniques such as MR Spectroscopy and Diffusion Tensor Imaging to objectively classify subjects based on disease severity and predict adverse outcomes.

**Methods:**

41 cases of neonatal encephalopathy with MRI Brain were included in the study. NAA/Cr, NAA/Cho, Cho/Cr and Lac/Cr ratios in basal ganglia and white matter, and DTI parameters of FA, ADC and MD in five major tracts were recorded. Developmental milestones were assessed on follow up of the child at 6 months. Correlation of the MRS and DTI values with disease severity and status at follow up was done.

**Results:**

The ratios of NAA/Cr and Lac/Cr in basal ganglia and white matter showed strong positive correlation with the disease severity at onset. Lac/Cr also positively correlated with the abnormal outcome group. FA values in the posterior limbs of internal capsule, thalami and corpus callosum were seen to most consistently correlate with the abnormal outcome group, substantiating these objective and reproducible metrics that can be employed for neuro-prognostication across all etiologies of neonatal encephalopathy.

**Conclusion:**

Neonatal encephalopathy is an umbrella term for conditions that can cause devastating neurological sequelae, which necessitates radiological pattern recognition for identification of etiology, and use of supplementary quantitative techniques such as MRS and DTI is endorsed for objective evaluation of clinical outcomes.

## Introduction

Neonatal encephalopathy (NE) is a broad term for a neurological condition occurring within the early neonatal period, characterized by symptoms such as seizures, impaired consciousness, reduced muscle tone and reflexes, respiratory difficulties, and in some cases, involvement of multiple organ systems.^1^ The underlying causes of NE are diverse and most commonly include hypoxic-ischemic encephalopathy (HIE) due to perinatal asphyxia. Metabolic disturbances also play a significant role in the development of neonatal encephalopathy. One such common condition is neonatal hypoglycemia, and newborns with hypoxic-ischemic encephalopathy (HIE) are especially susceptible to coexisting hypoglycemia, which can intensify the neurological injury.^2,3^ Infectious causes like viral leukoencephalopathies are also common culprits of NE. Many viruses show a distinctive pattern of white matter injury, characterized by symmetrical diffusion restriction in the periventricular white matter and major tracts, like the corpus callosum.^4–7^ Other causes can be coagulation abnormalities, genetic disorders, congenital infections, inborn errors of metabolism, and severe birth-related injuries.^2^

Relying on clinical evaluation alone is insufficient in providing a comprehensive understanding of the etiology, diagnosis, or prognosis of the condition. Neuroimaging, particularly advanced magnetic resonance imaging (MRI) techniques, presently assumes a pivotal role in pointing towards a definite etiology, assessing the extent of cerebral injury, providing prognostic insights as well as an understanding of the temporal dynamics of the injury. Although ultrasonography (USG) remains the primary imaging modality for the assessment of the neonatal brain, particularly in preterm infants, due to its portability and accessibility- MRI is the gold standard due to the high sensitivity and specificity, coupled with its capability for functional imaging.^4^

Conventional MRI mainly aids in identification of imaging patterns thereby narrowing differential diagnoses. Advanced MR techniques, including but not limited to MR Spectroscopy (MRS) and Diffusion Weighted Imaging (DWI) are especially useful in the acute phase for accurate estimation of extent and locations of cerebral injury.^8^

Proton magnetic resonance spectroscopy (MRS) enables the non-invasive, in vivo measurement of brain metabolites in specific regions, providing insight into the altered biochemical processes in NE.^5,9,10^ Key metabolites include N-acetylaspartate (NAA), which is a marker of structural integrity localized to neurons, and Lactate, which accumulates as a byproduct of anaerobic glycolysis. The neurometabolic data provided by these have demonstrated significant prognostic value in neonates with asphyxia, where reduced NAA levels and elevated lactate concentrations are associated with a poor neurological outcome.^11^ However, the absolute signal intensity of metabolites can vary due to a number of factors like scanner calibration, coil loading, magnetic field inhomogeneity, voxel placement, and patient factors. Ratios can normalize for these variations. Creatinine (Cr) is noted to be most stable across brain regions and pathological conditions; and using it as a denominatot can allow simple clinical application of the ratios without requiring complex quantification methods like water referencing or absolute concentration calculations.

Other techniques like Diffusion Tensor Imaging (DTI) have also been studied for their ability to quantify white matter injury, thereby aiding in neuro-prognostication in specific developmental domains.^12^ Diffusion Tensor Imaging (DTI) produces various scalar metrics—such as fractional anisotropy (FA) and mean diffusivity (MD)—which reflect how water diffuses through brain tissue. Autopsy findings have shown that brain myelination occurs in a caudo-cranial direction and at varying rates across different brain areas. Motor and sensory tracts tend to mature earlier than higher-order association pathways.^13,14^ Diffusion MRI exploits the directional properties of water movement—diffusing more easily along the length of axons and encountering resistance perpendicular to them due to barriers such as myelin and cell membranes. This makes it useful for evaluating the integrity and organization of white matter fiber tracts and identifying abnormalities, as it is influenced by factors like cellular architecture, fiber orientation, and cell density.^15,16^ Changes such as cytotoxic or vasogenic edema, inflammation, and neuronal loss, commonly seen in neonatal encephalopathy, can be effectively detected using these measures.^12^

FA reflects the degree to which diffusion is directionally restricted, while ADC measures the overall magnitude of water diffusion. Both metrics offer valuable insights into white matter development and structural integrity. Previous studies have linked poor early neurological outcomes in neonates with hypoxic-ischemic encephalopathy (HIE) to decreased FA and increased ADC values in specific white and gray matter regions.^16^

Our study aims to explore the various pathologies that are encompassed by the umbrella term NE, and the specific imaging patterns on MRI which can indicate the diagnosis thereby influencing the treatment patterns at an early stage. Beyond this, application of quantifiable and reproducible measures of injury like MRS and DTI is studied to classify the severity of disease and role in neuro-prognostication.^17^

## Methodology

This was a prospective observational study conducted in the Department of Radiodiagnosis & Imaging, Kasturba Medical College and Hospital, Manipal between 7th June 2023 and 31st January 2025. Independent Institutional Ethics Committee approval was obtained (number IEC 266/2023) and study was registered with the Clinical Trials Registry Of India (CTRI/2023/07/055568). Informed consent was obtained from the guardians of all participants of the study.

41 cases aged 0–30 days (including 10 late preterm GA infants between 36 and 37 weeks) referred to Department of Radiodiagnosis for MRI Brain, with clinical diagnosis of encephalopathy were included, due to perinatal hypoxia/other etiologies. This included neonates showing any of 3 symptoms-altered mental sensorium, recurrent seizures, or abnormal neurological function. Neonates with severe congenital or chromosomal anomalies, those unwilling to give consent and participate, and excessively degraded images were excluded.

### Image acquisition and assessment

Under adequate sedation (syrup Pedicloryl; Chloral hydrate 0.25–0.5 mL/kg body weight) administered by the neonatologist, MRI Brain was performed on United uMR780 3T MRI using dedicated neonatal head coil. Standard sequences acquired were- Axial and Sagittal T1, Axial T2 and T2 FLAIR, DWI images with b value 0 and 1000 with imaging angle along orbitomeatal line and a 25 cm field of view.

For MR spectroscopy, Axial T2 localizer was used and grid was applied (thickness 5 mm, gap 0. 5 mm, FOV 180 × 180, Matrix 336 × 336); 4 separate voxels (1.5 × 1.5 mm^3^) were placed in two regions of interest at both sides; the basal ganglia and parieto-occipital white matter.

**Figure 1.**
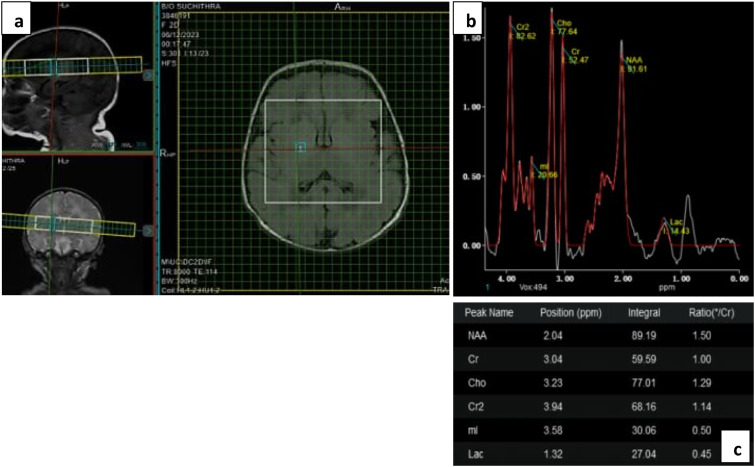
(a) shows voxel placement in left basal ganglia on short TE (35) with (b) metabolites graph and (c) values of NAA, Cho, Cr and Lac.

Contact with the cerebrospinal fluid and skull bone was avoided in voxel placement. Localized shimming, phase correction were performed prior to acquisition of the spectra. The pulse sequence used was point resolved spectroscopy (PRESS) with parameters; long TE (TR/TE = 1500/144 ms) and short TE (TR/TE = 1500/35 ms). Typical acquisition time was 3 min 30 s per spectral acquisition; 7 min for total scan. Long TE was used to visualize intensity peaks of NAA (2.02 ppm), Cho (3.22 ppm) and Cr (3.04 ppm) and to calculate NAA/Cr, NAA/Cho and Cho/Cr ratios. Short TE was mainly used to illustrate Lac (1.29 ppm) peak and to calculate Lac/Cr ratio (Figure 1).

**Figure 2.**
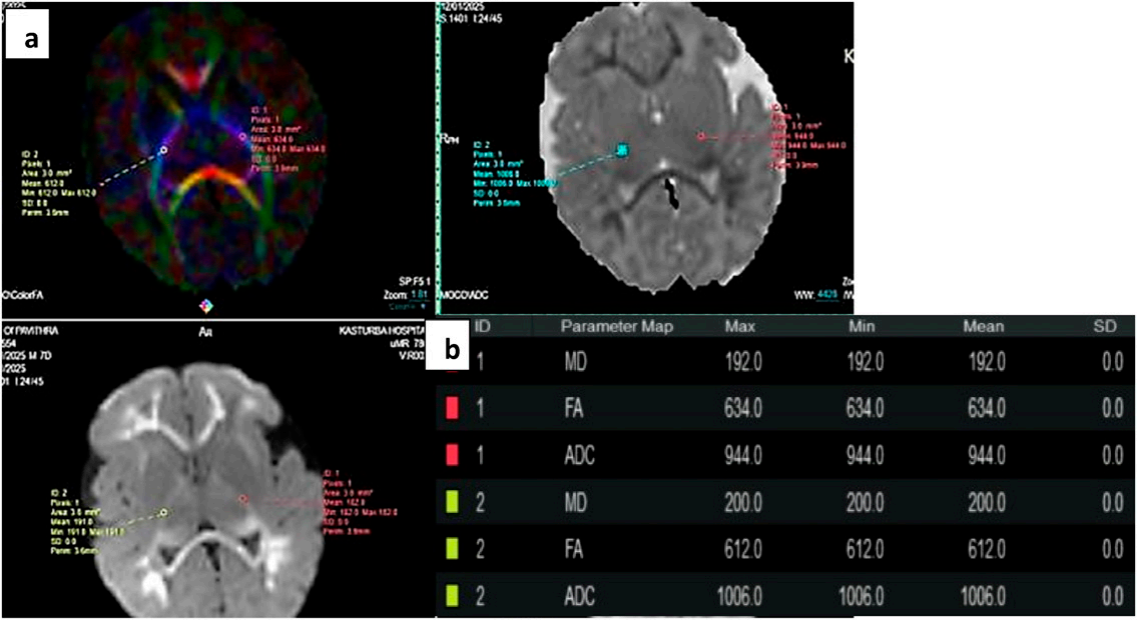
(a) shows ROI placement in the posterior limbs of internal capsules in Color FA maps bilaterally with corresponding DWI and ADC maps. (b) shows the FA, ADC and MD values in the ROIs.

The diffusion tensor sequence was performed by a single shot echo planar (EP) sequence using 32 non-collinear directions of the diffusion gradients using the following parameters: TR = 5625 ms, TE = 82 ms, slice thickness = 4 mm with no gap, FOV = 25 cm, matrix of 10 and b value = 1000 s/mm^2^. The DTI sequence lasted 6 min 30 s (Figure 2).

Data was post processed at United MR Workstation and parametrics of FA, ADC and MD were acquired using circular Regions Of Interest (ROIs) of fixed size (area 3 mm^2^ and 1 pixel) in 5 areas bilaterally - Posterior Limbs of Internal Capsule, Thalami, Corpus Callosum (Genu and Splenium), Deep White Matter of Frontal Lobes (corresponding to Inferior Fronto-Occipital Fasciculus), and Parietal Lobes (corresponding to Superior Longitudinal Fasciculus).

The relevant clinical history including the delivery history of the patient was recorded, along with the clinical grading of the encephalopathy as provided by the neonatologist, according to Sarnat & Sarnat/Thompson grading in cases of HIE. Other cases of encephalopathy were classified based on severity into Mild, Moderate and Severe categories considering the EEG findings, requirement and duration of respiratory support, and clinical course of the child.

The clinical follow up of the child was obtained 6 months after scan (with up to 2 weeks variation for corrected GA of pre-term infants) at the High Risk Neuro-Developmental Follow Up OPD at Kasturba Hospital, and allotted an outcome of Normal/ Mild Developmental Delay/ Global Developmental Delay/ Death- based on personal-social milestones, weight gain, auditory and visual examination, plus fine and gross motor skills (based on Denver Developmental Scale).

### Statistical analysis

The qualitative data was numerically encoded, and along with the ratios and DTI values entered into MS Excel spreadsheet. Data was analyzed using the statistical package SPSS 26.0 (SPSS Inc., Chicago, IL) and integration using Python where relevant. Level of significance was set at *p* < 0.05. Inferential statistics was done using Independent T test (2 groups) and One way Anova test with Bonferroni posthoc test (3 groups). Chi square test was used for association of categorical variables. ROC curve was used to estimate predictive power of variables and generate optimal threshold values.

## Results

### Demographics

All subjects were aged <30 days at time of scan acquisition. There was a male predilection in the cases and majority of the cases were full term gestational age (76%; 31 cases). 10 cases were late preterm with gestational age between 36 and 37 weeks. Imaging was done once the child was stabilized as per institutional protocol, and was noted to be between days 3 and 10 relative to the presenting event, that is, the subacute phase of disease. Based on etiology, the major cohort was formed by cases of HIE (46%; 19 cases), out of which predominantly grade II cases were imaged. Rotaviral encephalopathy constituted another big group of cases (24%; 10 cases), and 5 neonates manifested with encephalopathy due to hypoglycemia. Other minor groups were metabolic, bilirubinemic and epileptic encephalopathies. Close to half the total number of cases (46%) showed mild severity of disease. 24% cases showed some lag in development based on the Denver Developmental screening test, and observations like paucity of limb movements and hypotonia, abnormal Oto-Acoustic Emissions tests, absence of social smile, inadequate weight gain, persistent Moro’s reflex and microcephaly. 3 cases showed multiple abnormal findings with significant or global delay in development. There was no follow up available for 5 cases, and no death was observed within our group (Table 1). The correlation between clinical severity and follow up was statistically significant (*p* 0.018), indicating that more severe cases at onset presented with delayed milestones.

**Table 1.** Demographic and clinical details of study subjects.

		Frequency (N = 41)	Percentage (%)
Gender	Male	26	63.4
	Female	15	36.6
Birth history	Term	31	75.6
	Preterm (GA 36–37 weeks)	10	24.3
Mode of delivery	Normal vaginal delivery	22	53.6
	LSCS	19	46.3
Etiology	HIE grade I	4	9.8
	HIE grade II	14	34.1
	HIE grade III	1	2.4
	Rotaviral	10	24.4
	Hypoglycemic	5	12.2
	Hyperbilirubinemic	2	4.9
	Metabolic	3	7.3
	Epileptic	1	2.4
	Viral leukoencephalopathy	1	2.4
Clinical severity	Mild	19	46.3
	Moderate	11	26.8
	Severe	11	26.8
Clinical follow up	Normal	23	56.1
	Mild developmental delay	10	24.4
	Global developmental delay	3	7.3
	Death	0	0
	Not available	5	12.2

GA- gestational age, LSCS- lower segment caesarean section, HIE- hypoxic-ischemic encephalopathy.

### MRI Parameters

**Figure 3.**
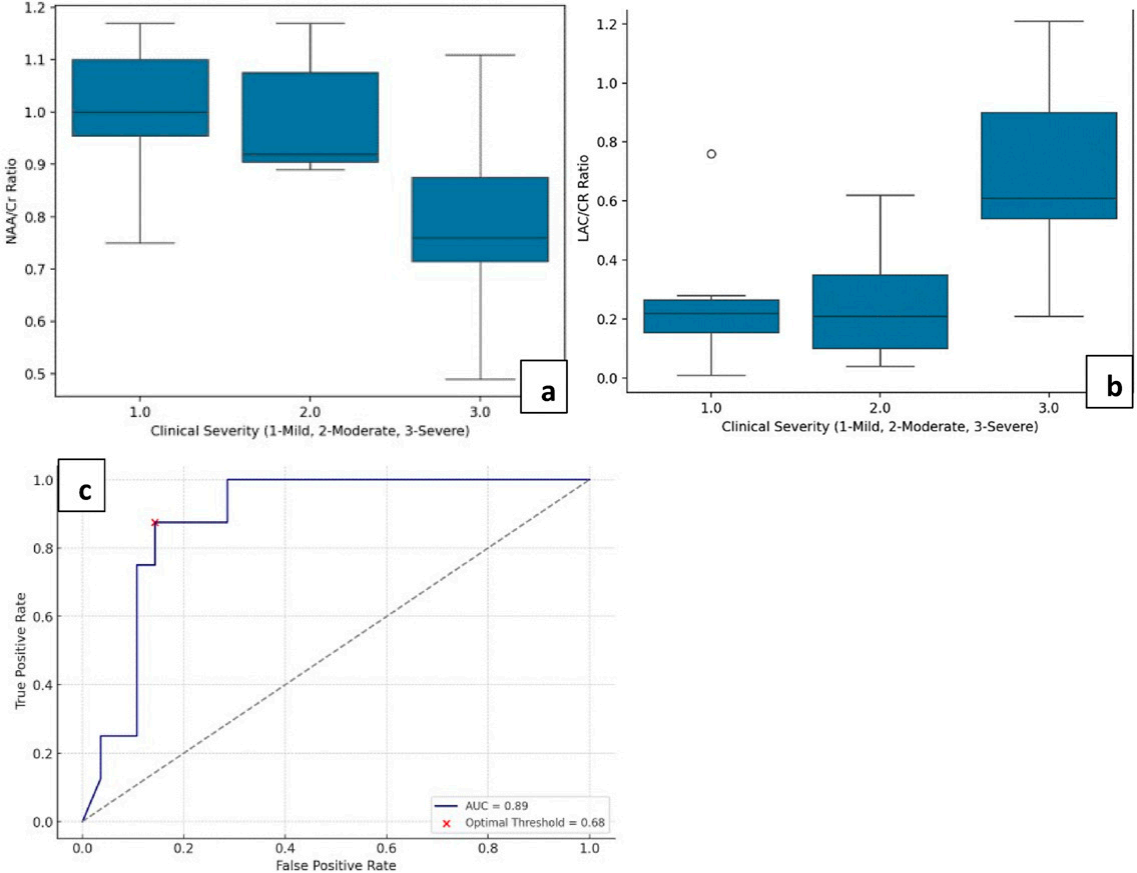
(a) and (b) show boxplot charts of NAA/Cr and Lac/Cr ratios across clinical severity groups, respectively. (c) is the AUC curve for double outcome model of Lac/Cr as a predictor for higher disease severity and developmental delay.

MRS ratios in the basal ganglia and parieto-occipital white matter, compared across mild, moderate, and severe groups showed significant difference amongst groups for NAA/Cr and Lac/Cr. In basal ganglia, there was a significant difference in values between the groups (p = 0.001*), with the severe group showing the lowest mean ratio of NAA/Cr (0.7945); and higher mean ratio of Lac/Cr (0.70) (Table 2).Similarly, in parieto-occipital white matter, the severe group had significantly lower NAA/Cr ratios (0.6918), and higher Lac/Cr values (0.630; *p* = 0.001*) compared to mild and moderate groups (Figure 3). On comparison of NAA/Cho and Cho/Cr in both ROIs across clinical severity groups, no statistically significant difference was observed in values in both locations (*p* > 0.05).

**Table 2.** MR Spectroscopy ratios in basal ganglia and parieto-occipital white matter.

	Mild (19)	Moderate (11)	Severe (11)	*p*- value
Basal ganglia				
NAA/Cr	1.04 ± 0.15	0.98 ± 0.10	0.79 ± 0.16	0.001*
NAA/Cho	0.59 ± 0.13	0.60 ± 0.07	0.50 ± 0.12	0.07
Cho/Cr	1.69 ± 0.25	1.62 ± 0.13	1.67 ± 0.19	0.64
Lac/Cr	0.22 ± 0.15	0.24 ± 0.18	0.70 ± 0.33	0.001*
Parieto-occipital white matter				
NAA/Cr	1.13 ± 0.38	1.23 ± 0.22	0.69 ± 0.22	0.001*
NAA/Cho	0.79 ± 0.38	0.73 ± 0.22	0.56 ± 0.20	0.14
Cho/Cr	1.67 ± 0.54	1.80 ± 0.41	1.53 ± 0.45	0.77
Lac/Cr	0.43 ± 0.36	0.48 ± 0.42	1.11 ± 0.38	0.001*

NAA- N-acetyl aspartate, Cho- choline, Cr- creatinine, Lac- lactate. p values < 0.05 indicating significance of data have been highlighted in bold.

MRS ratios were also compared across the broad clinical follow up groups of normal and delayed development, and statistically significant difference in values was only seen in Lac/Cr ratios measured in the parieto-occipital white matter. ROC analysis was generated for Lac/Cr ratios based on a double outcome model of predicting higher disease severity and developmental delay (Figure 3), and an optimal threshold value of ∼0.685 was obtained with specificity of 85.7% in our demographic. This combined outcome model for Lac/Cr in white matter showed to be a strong biomarker for identifying high-risk patients.

The DTI metrics in all 10 ROIs in brain were compared across the normal and delayed developmental outcome groups to assess the prognostication power. FA was seen to be the strongest parameter, with statistically significant correlation seen across groups in both posterior limbs of internal capsule and thalami bilaterally, and both genu and splenium of corpus callosum (Table 3). No significant difference in values was observed in the tracts in frontal and parietal white matter.

**Table 3.** Mean FA values in 10 ROIs (5 tracts bilaterally).

Mean FA values in major WM tracts bilaterally		Normal (23)	Delayed development (13)	*p* value
Posterior limbs of internal capsule	Left	568.65 ± 90.15	307.31 ± 78.65	0.001*
	Right	568.78 ± 98.04	307.62 ± 50.34	
Corpus callosum	Genu	525.61 ± 146.04	398.00 ± 107.20	0.009*
	Splenium	606.83 ± 88.81	414.92 ± 131.29	0.001*
Thalami	Left	213.78 ± 39.44	140.62 ± 23.66	0.001*
	Right	228.00 ± 26.85	146.00 ± 22.28	
Inferior fronto-occipital fasciculus	Left	148.35 ± 57.87	160.54 ± 47.48	0.52
	Right	146.78 ± 60.03	163.00 ± 45.62	0.40
Superior longitudinal fasciculus	Left	172.96 ± 80.92	205.31 ± 10.30	0.30
	Right	167.26 ± 79.91	182.92 ± 97.43	0.60

FA- fractional anisotropy, WM- white matter, ROI- regions of interest. p values < 0.05 indicating significance of data have been highlighted in bold.

**Figure 4.**
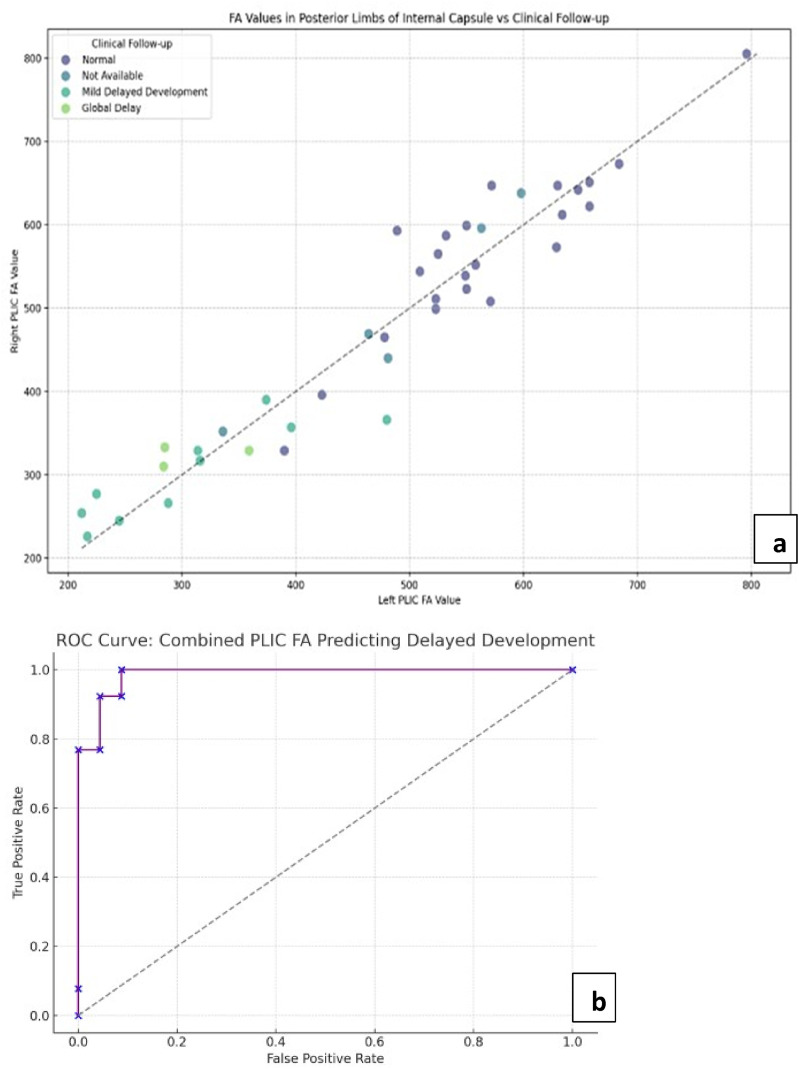
(a) is the scatter plot for combined FA values in PLIC across clinical follow up groups of normal and delayed development, showing lower values in cases of delayed development. (b) is the AUC curve showing remarkable predictive power of FA in PLIC at threshold of 413 in the study’s demographic.

ROC analysis using FA as a consistent marker in PLIC generated an optimum threshold of ∼ 413.0 to predict abnormal outcome with an excellent specificity of up to 87% (Figure 4). Threshold values were also generated in the Thalami (∼182) and Genu and Splenium of Corpus Callosum (∼527 and ∼520, respectively) with comparable sensitivity and specificity.

**Figure 5.**
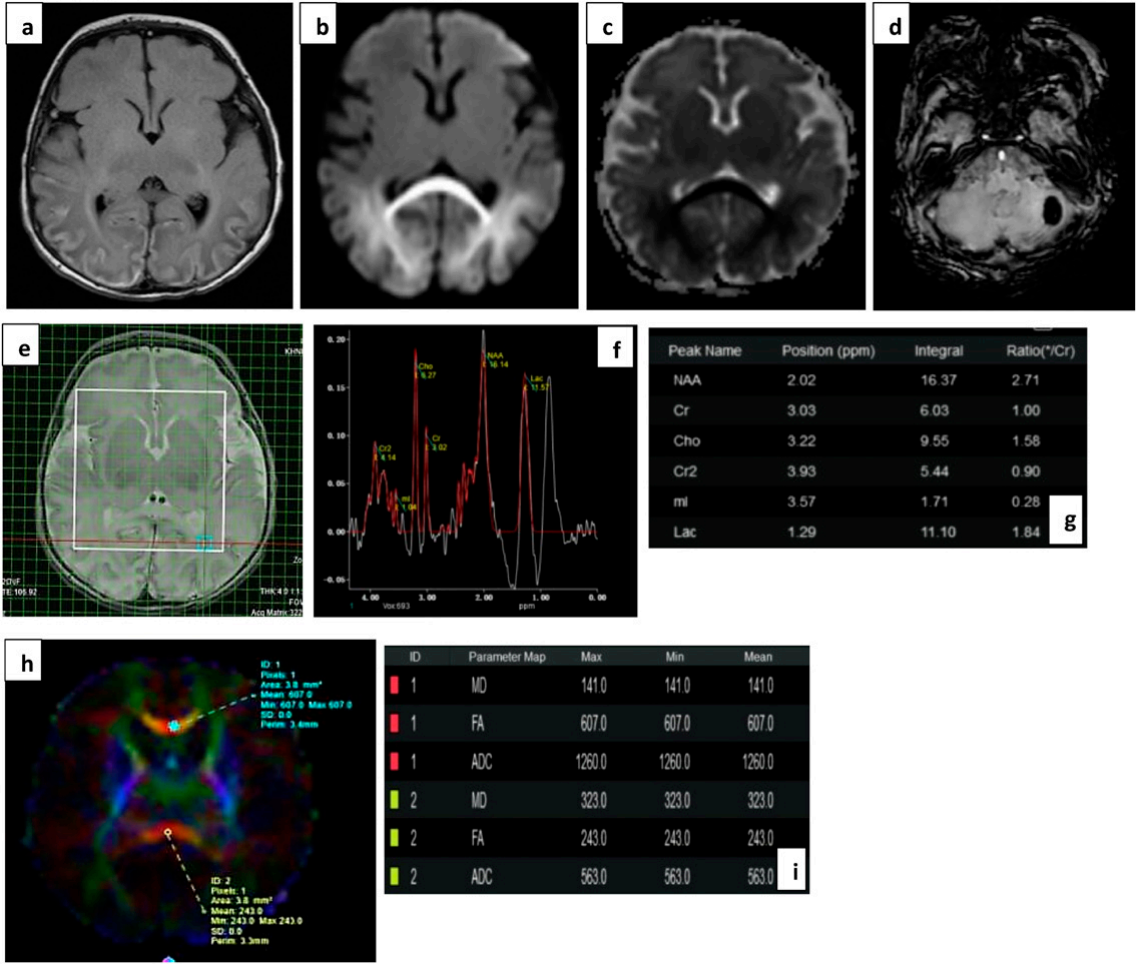
(a) T2 FLAIR, (b) and (c) DWI images show FLAIR hyperintensity with diffusion restriction in the parieto-occipital white matter and splenium in a severely sick infant with hypoglycemia. (d) SWI Images show a haemorrhagic focus in left cerebellar hemisphere. (e), (f) and (g) show voxel placement with graph and values in left POWM. Lac peak with elevated values (Lac/Cr- 1.84). (h) and (i) show ROI in the genu and splenium of corpus callosum with markedly reduced FA values in splenium, the involved part. The neonate showed mild delay in achievement of milestones.

**Figure 6.**
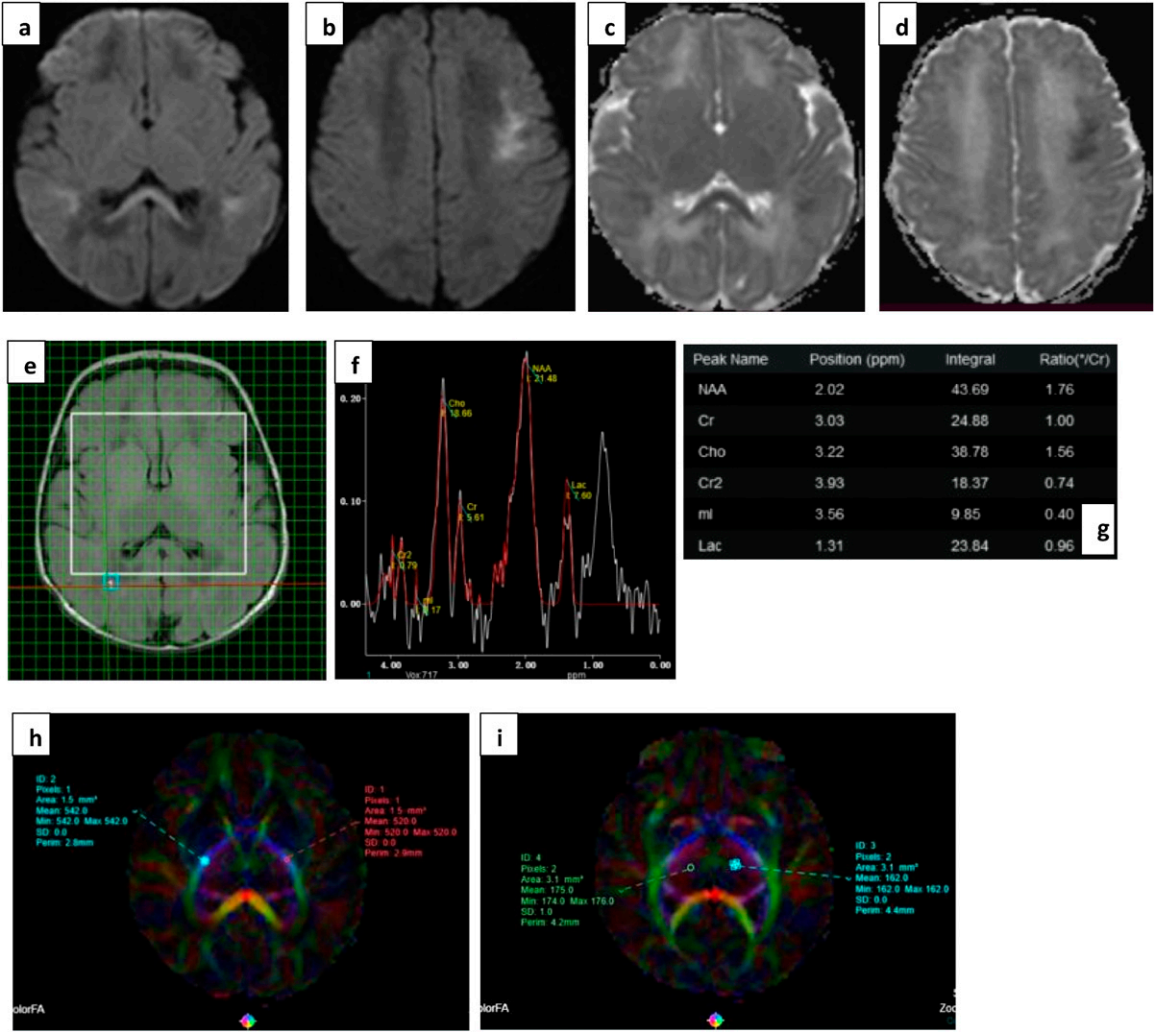
(a), (b) DWI and (c), (d) ADC images show true diffusion restriction in splenium, periventricular and subcortical white matter (watershed injury) in a preterm infant with grade II HIE. (e), (f) and (g) show voxel placement with graph and values in right POWM. Lac peak is noted with increased Lac/Cr ratio of 0.96. (h) and (i) images show ROI placement in PLIC and Thalami with borderline normal values. Neonate showed normal milestones on follow up.

**Figure 7.**
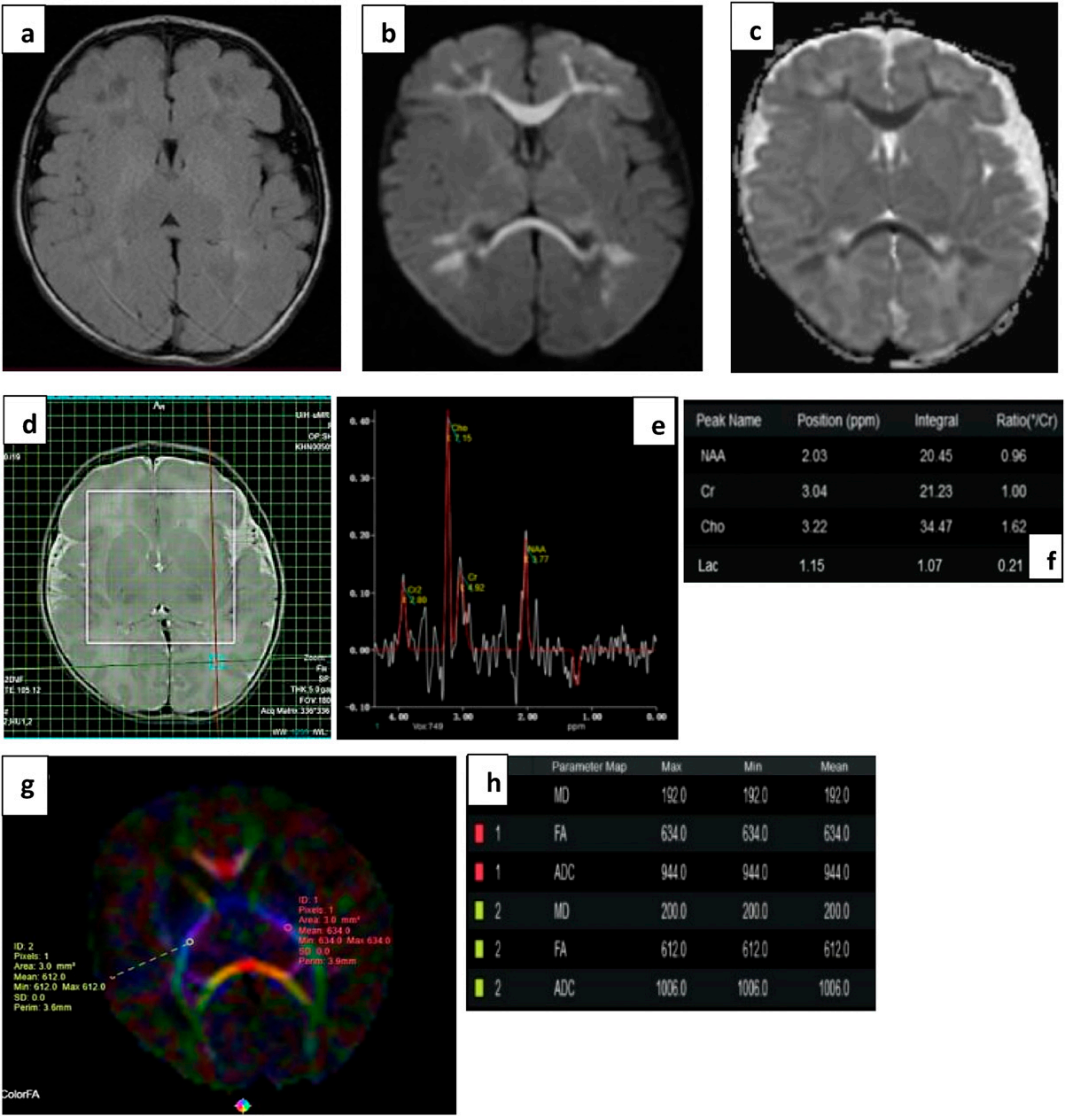
(a) T2 FLAIR, (b) DWI and (c) ADC images subtle FLAIR hyperintensities with true diffusion restriction symmetrically involving the genu and splenium of corpus callosum, and periventricular and subcortical white matter bilaterally, typical pattern for viral encephalitis. The rotavirus stool RNA was positive in this neonate. (d), (e), and (f) images show voxel placement with graph and values in right POWM. Normal Lac values without any abnormal metabolite peak seen. (g) and (h) show ROI placement in PLIC with normal FA values. This infant showed normal milestones on follow up.

A difference in ADC values between normal and abnormal outcome groups was only seen in the corpus callosum, and inconsistently in the thalamic tracts (Table 4). MD showed significant difference across groups only in the genu of corpus callosum; however, inconsistency of values within the parts of corpus callosum itself renders it an unreliable marker (Table 5) and Figures 5, 6 and 7.

**Table 4.** Mean ADC values in 10 ROIs (5 tracts bilaterally).

Mean ADC values in major WM tracts bilaterally		Normal (23)	Delayed development (13)	*p*- value
Posterior limbs of internal capsule	Left	973.96 ± 85.25	1004.69 ± 84.49	0.30
	Right	993.13 ± 99.92	1047.77 ± 114.56	0.14
Corpus callosum	Genu	1024.61 ± 34.38	1388.08 ± 24.24	0.002*
	Splenium	881.96 ± 35.87	1303.15 ± 44.60	0.004*
Thalami	Left	1046.13 ± 10.56	1102.92 ± 13.61	0.17
	Right	1105.48 ± 86.56	1081 ± 10.04	0.02*
Inferior fronto-occipital fasciculus	Left	1492.65 ± 29.35	1502.08 ± 18.17	0.91
	Right	1400.04 ± 25.40	1441.08 ± 19.75	0.61
Superior longitudinal fasciculus	Left	1446.00 ± 37.98	1477.31 ± 27.73	0.79
	Right	1380.61 ± 238.98	1298.85 ± 20.29	0.30

ADC- apparent diffusion coefficient, WM- white matter, ROI- regions of interest. p values < 0.05 indicating significance of data have been highlighted in bold.

**Table 5.** Mean MD values in 10 ROIs (5 tracts bilaterally).

Mean MD values in major WM tracts bilaterally		Normal (23)	Delayed development (13)	*p*- value
Posterior limbs of internal capsule	Left	141.78 ± 34.62	146.08 ± 44.95	0.75
	Right	140.96 ± 35.30	147.31 ± 74.64	0.73
Corpus callosum	Genu	181.48 ± 97.51	117.92 ± 27.92	0.02*
	Splenium	205.30 ± 11.04	166.85 ± 13.65	0.36
Thalami	Left	130.13 ± 37.45	134.00 ± 34.50	0.76
	Right	130.65 ± 35.81	134.08 ± 34.79	0.78
Inferior fronto-occipital fasciculus	Left	121.87 ± 52.02	117.77 ± 24.32	0.71
	Right	136.52 ± 53.35	128.92 ± 29.71	0.64
Superior longitudinal fasciculus	Left	162.43 ± 10.18	151.77 ± 64.20	0.73
	Right	167.13 ± 87.65	174.54 ± 85.94	0.80

MD- mean diffusivity, WM- white matter, ROI- regions of interest. p values < 0.05 indicating significance of data have been highlighted in bold.

## Discussion

The earliest and most sensitive imaging marker of our biggest case cohort, hypoxic-ischemic encephalopathy, is diffusion restriction - the pattern of which can vary based on the severity and duration of asphyxia.^8,18–20^

In mild cases, injury is usually seen at the watershed areas of subcortical white matter. With increasing insult and disease severity, loss of cerebral autoregulation is seen with injury to higher metabolic areas like basal ganglia and thalami.^21,22^ These findings described by Varghese et al.^23^ are consistent in our study. Of the 4 mild cases, 2 cases showed injury at watershed zones, one being late preterm with periventricular lesions, and the other being term showing subcortical lesions. Of the 14 moderate HIE cases, 5 showed normal imaging and DTI parameters, with normal outcome. This can also be attributed to the timing of imaging relative to the presenting event. Imaging was limited in the acute phase of disease owing to the unstable neurological status of the child. In our study, imaging in cases of HIE was done between days 3 and 10 relative to the presenting event, that is, the subacute phase of disease. This lead to a variation in disease pattern observed on conventional MRI, as described.

Other cases of moderate HIE showed varying lesions in watershed areas and deep gray matter. Two cases showed mild developmental delay and both of these showed abnormal spectroscopy and DTI values. One case of severe HIE showed normal imaging with few micro-hemorrhages, which is likely related to delayed timing of imaging [day 12]. Notably, DTI parameters were abnormal with markedly reduced FA, and showed mild developmental delay. Such a reduction in values could be seen in some severe cases even when conventional MR imaging had normalized, or even in cases like hyperbilirubinemia which did not show any imaging findings.

Almost all cases of Rotaviral Encephalopathy (10) in our study showed mild clinical severity, with consequent normal clinical outcome. One case with concomitant hyperibilirubinemia and Atrial septal defect showed severe delay, with expected reduction in FA values in PLIC and CC. On conventional imaging, 9 of 10 show a typical ‘butterfly’ type pattern of injury with diffuse involvement of periventricular and subcortical white matter, corpus callosum, and characteristic deep gray matter sparing.^6,24,25^ Another case of viral encephalitis with such typical involvement showing multiple micro-hemorrhages was noted to be positive for varicella, indicating characteristic vascular involvement. The MRS and DTI parameters were seen to be normal in these cases.^26,27^

Common metabolic causes like hypoglycemia show characteristic predilection for parieto-occipital white matter, as described by Boardman et al.^23^ 5 cases of hypoglycemic encephalopathy in our study showed severe clinical presentation with 2 cases developing mild delay in milestone achievement. Remarkably, both of these cases showed normal MR imaging, however with markedly elevated lactate levels in the parieto-occipital white matter. Rarely, severe cases can also show intra-parenchymal haemorrhages—which was observed in one case in our study.^28^ Cases of bilirubin encephalopathy typically show T1 hyperintensity in the globus pallidus; however, cases in our study showed subtle to no findings and this was a clinical diagnosis.^29,30^ This reflects the varying imaging findings found in this condition based on the duration and severity. Few other cases of metabolic encephalopathy were observed with derangements like hypocalcemia, however, showing normal MR imaging and parameters. One case of hypernatremic dehydration showed a diffusion pattern closely mimicking that of severe HIE.^31^ Child recovered on correction and normal follow up was noted. Few cases, like that of epileptic encephalopathy, showed no other abnormalities apart from minor subdural collections and few micro-hemorrhages.^32^

Few broad observations were made about the biomarkers studied, based on the etiology of encephalopathy. The cases of viral encephalopathy were seen to cause transient abnormalities with near normal values in all cases studied, by the time imaging was performed. The cases with available follow up also showed normal developmental outcomes. The cases with metabolic abnormalities like hypoglycemia and conditions like epileptic encephalopathy showed some of the lowest values in FA with subsequently delayed milestones on follow up. Lac/Cr was variable in these cases; and was seen to be close to normal values in cases with delayed imaging.

Our findings of significant correlation between NAA/Cr and Lac/Cr in both basal ganglia and white matter, and clinical severity of child agree with those of Elshal et al.,^33^ indicating MRS ratios of NAA/Cr and Lac/Cr are powerful diagnostic adjuncts. Similarly, Cho/Cr was found not significant across all studies.^34,35^ However, we did not find significance with NAA/Cho. Our findings also disagreed with Guo et al., who did not find any significance amongst ratios, and with Ancora et al.,^36^ who did not find Lac/Cr to correlate with clinical severity in basal ganglia (*p* > 0.05), where we found high significance. This can be attributed to the temporal variation of timing of scan with respect to the acute phase of insult; with a multitude of challenges delaying imaging-the availability of ventilatory support, requirement of sedation for scan duration, unstable vitals, etc. Some previous studies observed brain cooling can also lead to rise of Lac in gray matter, making values variable across time and treated cases. Two cases of moderate HIE in our study received therapeutic hypothermia, both of which showed elevated Lac/Cr levels, even so one case showed normalization of conventional MR findings.

Our study also did not find significant difference in NAA/Cho across clinical severity groups (*p* 0.07). Cho reflects the degree of dysmyelination and is usually elevated in neonates, showing steady decrease and varying values as myelination progresses. Further, Doormaal et al.^37^ hypothesized that Choline, being a marker for membrane synthesis, can show transient rise after insult owing to mobilization of membrane fragments. However, in subacute HIE, apoptosis has been seen to predominate, which preserves membrane integrity. This adds to the variability of Cho values and also supports the nil significant correlation of Cho/Cr across studies.

When compared with the clinical follow up of the child, our study found significant difference in values in Lac/Cr ratios in white matter, while other studies have variably and inconsistently found significance pertaining to NAA/Cr and NAA/Cho as well, predominantly in basal ganglia. It is likely that due to cerebral autoregulatory mechanisms, preferential clearance of byproducts of metabolism occurs in deep gray matter, and persistent elevation can be seen in other areas of the brain.

In the neonatal brain, MRS typically shows slightly lower N-acetylaspartate (NAA) levels, reflecting immature myelination. Following a perinatal brain injury, a transient drop in NAA indicates compromised neuronal function, while a rise in lactate suggests disrupted oxidative metabolism—likely due to mitochondrial dysfunction and increased activity of sodium-dependent transport mechanisms. These metabolite levels are dynamic and can change over time; consistently, a pattern of elevated lactate and reduced NAA strongly correlates with significant brain injury. Thayyil et al.^10^ observed that lactate elevation may persist well beyond the acute phase of injury. They proposed that this could result from ongoing metabolic disturbances in damaged brain tissue or from the accumulation of microglia, which rely on anaerobic glycolysis and contribute to sustained lactate production.^38^ Our study derived a threshold value of ∼0.685 for Lac/Cr in WM to predict both severity of disease and delayed outcome, with comparable sensitivity and specificity to Elshal et al., who found a cutoff of ∼0.42 for pathological outcome prediction.

Our study confirms the levels of Lac/Cr in white matter to be a reliable prognostic biomarker for neurodevelopmental outcome and derives a threshold value for our demographic.

Our study found significant difference in FA values in PLIC, Thalami and Corpus Callosum across normal and pathological outcome groups, in agreement with the findings of Li et al.^39^ and Dibble et al.,^40^ however, contradicting findings of Ancora et al. who did not find difference in groups for values in genu and splenium of corpus callosum. These findings are likely consistent as FA is a function of both axial and radial diffusivity with orthogonal sensitivity, making it highly sensitive to microstructural damage particularly in areas with dense white matter, where fibers are arranged closely and in parallel, and with higher volume of myelinated fibers-like the corpus callosum, and posterior limbs of the internal capsules.^17^ Our study derived a threshold of ∼0.413 of FA in PLIC, with 85% sensitivity and 87% specificity for prediction of abnormal neurodevelopmental follow up, comparable to the value derived by Li et al. of ≥0.395 predicting a good outcome with corresponding sensitivity and specificity- 70.2% and 79.5%, respectively.

No significant difference in any of the values was observed in the tracts in frontal and parietal white matter, likely owing to lack of myelinated tracts, incomplete neuronal maturation and lesser detection of fibers due to tensor acquisition in limited directions.

Our study also showed significant discriminatory power of FA values in PLIC across acute clinical severity groups. This is consistent with previous studies, as it is dependent on axonal membrane integrity which can change acutely. FA in PLIC is a consistent and reproducible marker of injury due to high myelination at birth and proves to be a powerful prognosticator in our study.

Previous studies found no significant differences in ADC values among the different groups, as these values are non-specific (no directional information) and important in early diagnosis of HIE, however, show pseudo-normalization after the acute stage (5–7 days) further decreasing its diagnostic power. However, our study similar to Kushwah et al.^16^ found significant difference in groups in ADC values in thalami, likely due to varying timing of scans and ADC being a better marker in more isotropic tissues, that is, with less directionality, like gray matter.

Ancora et al.^36^ reported meaningful differences in mean diffusivity (MD) values when comparing infants with normal versus adverse outcomes. Previous research highlights MD’s strong sensitivity to subtle structural disruptions, particularly within white matter tracts such as the corpus callosum, as was observed in our study in the genu of the corpus callosum. This limited observation may be explained by the temporal behaviour of MD—similar to ADC—where values initially rise and then undergo pseudo-normalization. As a result, the predictive reliability of MD measurements is highly dependent on when the imaging is performed.

This study has several limitations. It is a single center study with limited sample size, and without inter-observer and test-retest reliabilities-increasing the risk of false positives. Consideration of the effect sizes and confidence intervals is essential to determine the clinical utility. The sample size of the study is limited due to the time-bound design. Many of the cases qualifying for this study present acutely with distress and unstable vitals, requiring monitoring and largely restricting the sedation. Thereby acquisition of positive cases in acute stage is limited. Imaging is usually done once neonates are stabilized thereby parameters are varying based on time interval to scan; and this delay is not accounted for. This study also had a short follow up period with no imaging at subsequent visits, also attributed to the design of study, and the clinical follow up of the child has been assessed through a screening test. Therefore, all derived parameters should be interpreted as supportive and adjunctive, and as guiding indices rather than absolute prognostic markers and values, and a study with a longer follow up period and diagnostic tools can be conducted with validation in an independent dataset for more robust diagnostic validity. No control group was considered due to ethical reasons. Apart from acquisition, the processing of images proved challenging for voxel placement, due to small size of brain and overlap with cranium/CSF spaces.

## Conclusion

The varying etiologies of encephalopathy in the vulnerable neonatal group can cause lasting neurological damage. Not only is pattern recognition important for radiological diagnosis, analysis and application of quantitative and reproducible techniques is warranted for neuro-prognostication. MR Spectroscopy shows most consistent changes in NAA/Cr and Lac/Cr levels for predicting clinical severity of disease, and Lac/Cr values can be highly accurate in estimating the degree of injury, and therefore, the expected outcome of the child. FA has proven to be the most continuous and reproducible parameter in Diffusion Tensor Imaging, reflecting microstructural disruption particularly in packed and myelinated tracts like PLIC, and this can be used to prognosticate outcome based on tract involved.

## Appendix

### Abbreviations

ADC: Apparent diffusion coefficient
CC: Corpus callosum
DTI: Diffusion tensor imaging
DWI: Diffusion weighted imaging
FA: Fractional anisotropy
FLAIR: Fluid attenuation inversion recovery
GA: Gestational age
HIE: Hypoxic ischemic encephalopathy
MD: Mean diffusivity
MRI: Magnetic resonance imaging
MRS: Magnetic resonance spectroscopy
NAA: N-Acetyl aspartate
NE: Neonatal encephalopathy
PLIC: Posterior limbs of internal capsule
POWM: Parieto-occipital white matter
ROI: Regions of interest
USG: Ultrasonography
WM: White matter.
